# Spatial distribution and determinants of thyroid cancer incidence from 1999 to 2013 in Korea

**DOI:** 10.1038/s41598-021-00429-w

**Published:** 2021-11-18

**Authors:** Jieun Jang, Dae-Sung Yoo, Byung Chul Chun

**Affiliations:** 1 Department of Preventive Medicine, Korea University College of Medicine, Seoul, Korea; 2 Veterinary Epidemiology Division, Animal and Plant Quarantine Agency, Gimcheon, Korea

**Keywords:** Diseases, Health care, Risk factors

## Abstract

We evaluated the spatial variation in thyroid cancer incidence and its determinants in Korea considering its importance in cancer prevention and control. This study was based on the ecological design with cancer incidence data by administrative district from the National Cancer Center and regional characteristics generated from the Korea Community Health Survey Data. We identified spatial clusters of thyroid cancer incidences based on spatial scan statistics. Determinants of regional variation in thyroid cancer incidence were assessed using the Besag-York-Mollie model with integrated nested Laplace approximations. Spatial clusters for low and high thyroid cancer incidences were detected in the northeastern and southwestern regions, respectively. Regional variations in thyroid cancer incidence can be attributed to the prevalence of recipients of basic livelihood security (coefficient, − 1.59; 95% credible interval [CI], − 2.51 to − 0.67), high household income (coefficient, 0.53; 95% CI, 0.31 to 0.76), heavy smoking (coefficient, − 0.91; 95% CI, − 1.59 to − 0.23), thyroid dysfunction (coefficient, 3.24; 95% CI, 1.47 to 5.00), and thyroid cancer screening (coefficient, 0.38; 95% CI, 0.09 to 0.67). This study presented the spatial variations in thyroid cancer incidence, which can be explained by the prevalence of socioeconomic factors, thyroid cancer screening, thyroid dysfunction, and smoking.

## Introduction

Thyroid cancer is the most commonly diagnosed cancer in Korea. Its incidence is extremely high, especially among women (age-standardized incidence rates [ASR] in 2017: 36.8, 17.0, and 57.2 per 100,000 for total population, men, and women, respectively)^1^. The most notable epidemiological feature of thyroid cancer in Korea is the steep increase in the incidence rate^2,3^. In Korea, the thyroid cancer incidence rate increased from 10.7 to 57.2 per 100,000 among women and from 2.1 to 17.0 per 100,000 among men during the period 1999–2017^1^. Consequently, the economic burden due to thyroid cancer also increased substantially^4^.

Spatial disease cluster detection, a tool of spatial epidemiology, allows for identification and visualization of regions with abnormal disease incidence^5^. Identification of spatial inequality in cancer incidence based on the spatial cluster results in suitable designation of priorities, healthcare resource distribution, and medical policy implementation; therefore, spatial cluster detection is an efficient tool for cancer prevention and control.

Several studies have been conducted using this tool to assess regional variations in thyroid cancer incidence and to identify its determinants for health improvement^6–9^. However, these studies were limited in that analyses considering spatial autocorrelation were not performed, nor were major potential determinants that are well-known risk factors for thyroid cancer, such as radiation exposure^10^, obesity^11^, and thyroid conditions (such as autoimmune thyroiditis^12^ and goiter^13^), comprehensively considered.

In Korea, a study based on regional information of 16 administrative districts reported spatial variability in thyroid cancer incidence^14^. A positive correlation was also noted between the regional prevalence of thyroid cancer screening and regional thyroid cancer incidence rates. However, the limitations of the study were as follows: (1) district units considered to draw conclusions regarding regional variation in thyroid cancer incidence and its determinants were extremely large and (2) potential determinants except for thyroid cancer screening were not considered.

In South Korea, primary administrative district consists of 1 special city, 6 metropolitan cities, 8 provinces, 1 special self-governing province, and 1 special self-governing city. This primary administrative district is subdivided into municipal-level division (the second-level administrative district) including cities (si), counties (gun), and districts (gu). Currently, there are 229 municipalities (Sigungu districts) in South Korea. In 2016, the National Cancer Center in South Korea released statistics on regional cancer incidence in the Sigungu district for the first time^15^. A potential inequality in thyroid cancer incidence was reported in Korea (high incidence rate: most areas of Jeollanam-do and some metropolises such as Seoul, Daejeon, and Daegu; and low incidence rate: Gangwon-do). However, whether the region with a high or low thyroid cancer incidence rate is a significant disease cluster has not been assessed. In addition, although spatial analysis considering various potential determinants of regional variation in thyroid cancer incidence would be helpful in understanding the spatial epidemiology of thyroid cancer in Korea, exploration of these determinants has not been widely performed.

Therefore, we aimed to identify any significant spatial clusters of thyroid cancer incidence between 1999 and 2013 in Korea. Additionally, we investigated the determinants of the spatial variations in thyroid cancer incidence to better understand the epidemiological features of thyroid cancer in Korea.

## Results

**Figure 1:**
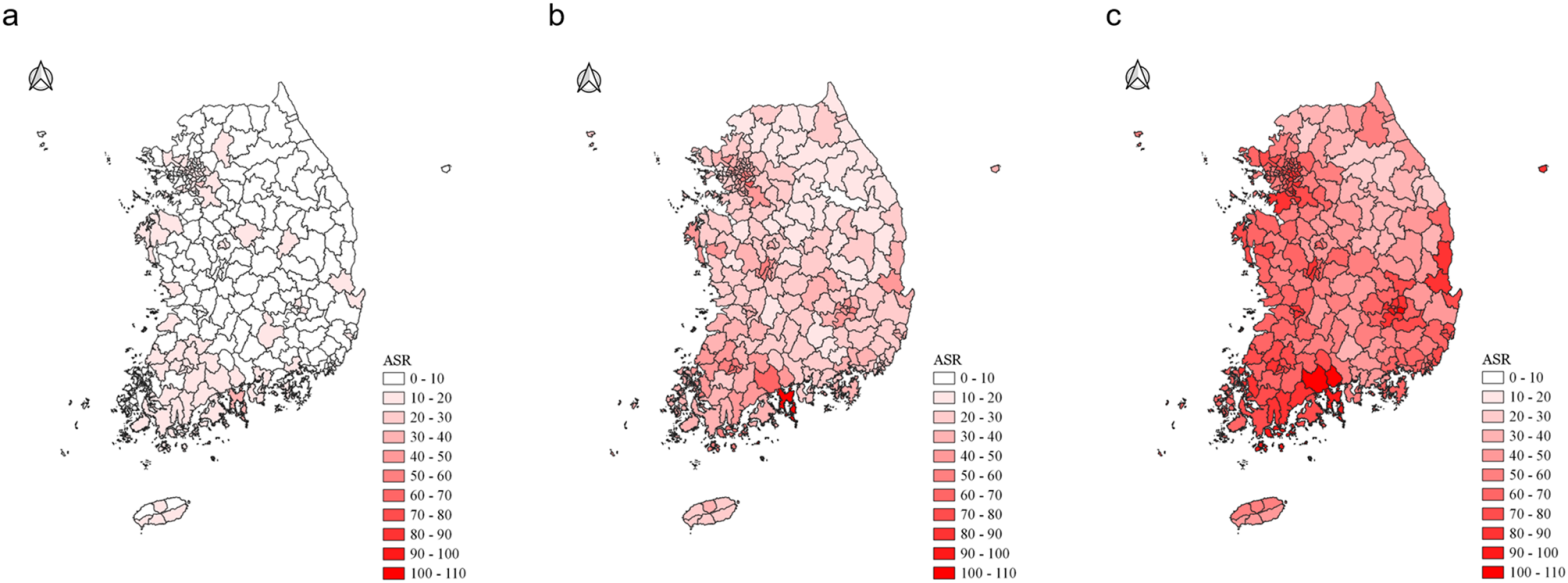
Choropleth maps for age-standardized incidence rates of thyroid cancer in Korea during (**a**) 1999–2003, (**b**) 2004–2008, and (**c**) 2009–2013.

The Sigungu district-level thyroid cancer incidence rates of three time periods (1999–2003, 2004–2008, and 2009–2013) were visualized on choropleth maps (Fig. 1). The incidence of thyroid cancer has been increasing rapidly across the country in recent years (ASR: 9.9/100,000 for 1999–2003; 32.4/100,000 for 2004–2008; 67.2/100,000 for 2009–2013). Thyroid cancer incidence rates appeared to be higher in areas located in the southwestern and northwestern regions of Korea than in other regions during the entire study period. In contrast, the Sigungu districts in the northeastern region were found to have a low ASR of thyroid cancer incidence.

**Figure 2:**
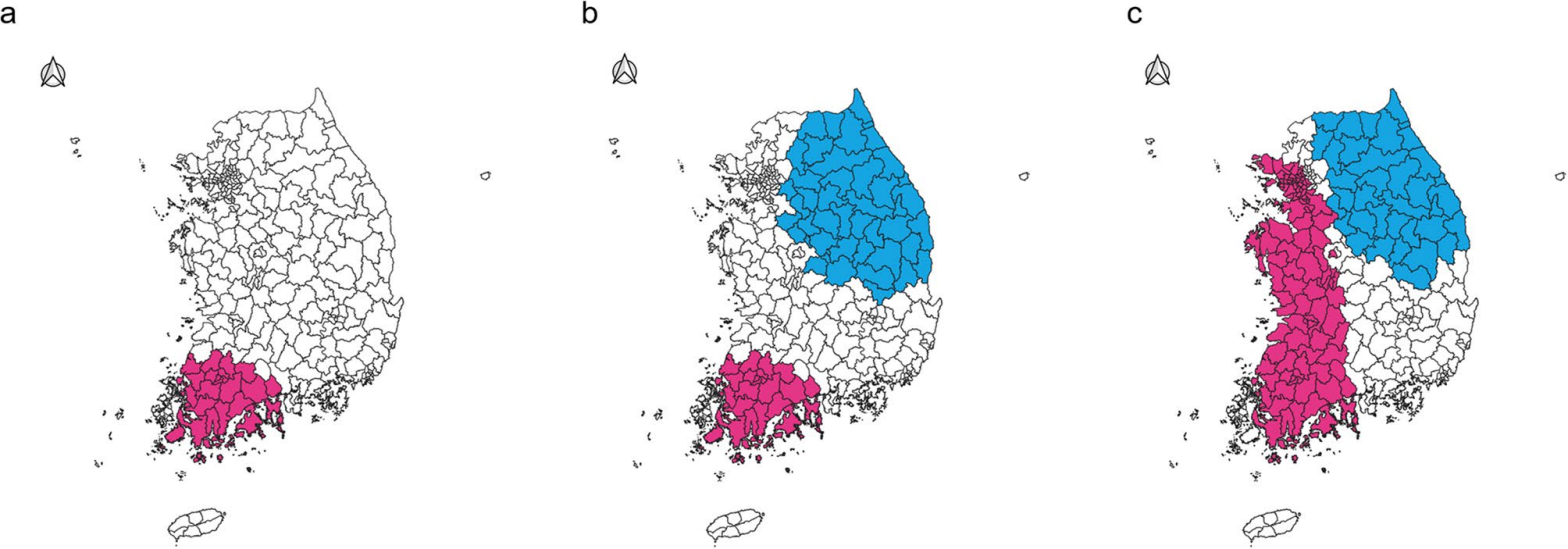
Spatial cluster for thyroid cancer incidence rates in Korea during (**a**) 1999–2003, (**b**) 2004–2008, and (**c**) 2009–2013. Red colored area in the southwest region and the blue colored area in the northeast region indicate spatial clusters with high and low thyroid cancer incidence rates, respectively.

We further assessed whether high or low thyroid cancer incidence rates in these regions were statistically different from those in other regions based on scan statistics (Fig. 2). The red colored area indicates a spatial cluster with a high thyroid cancer incidence rate (hot spot), whereas the blue colored area indicates spatial clusters with a low thyroid cancer incidence rate (cold spot). During the entire study period, spatial clusters with a significantly high thyroid cancer incidence rate were consistently observed in the southwestern region of Korea. In the latest study period (2009–2013), the thyroid cancer incidence rate was significantly higher in wide-ranging areas including the capital city and several metropolises than in other regions (ASR: 69.5/100,000 for inside the cluster and 56.3/100,000 for outside the cluster) (Table 1). On the contrary, the thyroid cancer incidence rate was significantly lower in the northeastern area than in other areas in Korea (ASR: 40.1/100,000 for inside the cluster and 66.0/100,000 for outside the cluster during 2004–2008; 17.9/100,000 for inside the cluster and 31.8/100,000 for outside the cluster during 2009–2013) during the two recent periods.

**Table 1:** Age-standardized incidence rates of thyroid cancer inside and outside the spatial clusters during 1999–2003, 2004–2008 and 2009–2013 in Korea.

Period	Inside the cluster1		Outside the cluster1		Inside the cluster2		Outside the cluster2		Total area
	N of districts	Incidence rate (N per 10^5^)	N of districts	Incidence rate (N per 10^5^)	N of districts	Incidence rate (N per 10^5^)	N of districts	Incidence rate (N per 10^5^)	Incidence rate (N per 10^5^)
1999–2003	25	14.41	222	8.6	-	-	-	-	9.9
2004–2008	25	47.5	222	27.3	43	17.9	204	31.8	32.4
2009–2013	110	69.5	137	56.3	46	40.1	201	66.0	67.2

*N* number.

We compared the regional characteristics between regions inside and outside the cluster for thyroid cancer incidence for the period 2009–2013 (Supplementary Table S1). The median prevalence of basic livelihood security recipients was significantly higher inside the cold spot (4.5%) than outside the cold spot (3.4%) (*P* < 0.01). The cold spot region had a low prevalence of high household income (5,000,000 won or approximately 4,600 dollars), whereas the prevalence of high household income was high in the hot spot region. Additionally, a high prevalence of heavy alcohol consumption and heavy smoking and a low prevalence of moderate or vigorous physical activity were observed in cold spots of thyroid cancer. On the contrary, we found low prevalence of heavy alcohol consumption and heavy smoking and high prevalence of physical activity in the hot spots of thyroid cancer. The prevalence of obesity was found to be higher in regions with low thyroid cancer incidence than in regions with a high incidence of thyroid cancer. Regions with high thyroid cancer incidence rates were found to have a significantly higher proportion of patients who underwent thyroid cancer screening. Though we hypothesized that external radiation would explain regional distributions of thyroid cancer incidence, we couldn't find significant association between the distance from nuclear power plant to centroid of each Sigungu district and regional thyroid cancer incidence rates.

Additionally, we used a Bayesian spatial regression model to investigate the determinants of regional variation in thyroid cancer with consideration of spatial autocorrelation on covariates and disease (Table 2). A 1% increase in the prevalence of basic livelihood security recipients was associated with a decrease of 1.6 per 100,000 in thyroid cancer incidence (coefficient, − 1.56; 95% CI, − 2.51 to − 0.67). On the contrary, one percent increase in prevalence of high household income (≥ 500,000 won) was associated with increase in 0.53 per 100,000 of the thyroid cancer incidence rate (coefficient, 0.53; 95% CI, 0.31 0.76). Elevation in one percent increase in proportion of heavy smokers was also associated with decline in about 1 per 100,000 of thyroid cancer incidence rate (coefficient, − 0.91; 95% CI, − 1.59 to − 0.23). Additionally, an increase in the prevalence of thyroid dysfunction and thyroid cancer screening was associated with increased thyroid cancer incidence rate (coefficient, 3.24; 95% CI, 1.47 to 5.00 for thyroid disorder; coefficient, 0.38; 95% CI, 0.09 to 0.67 for thyroid cancer screening). In other words, increase in 3.24 and 0.38 thyroid cancer incidences per 100,000 are linked to one percent increase in prevalence of thyroid dysfunction and thyroid cancer screening.

**Table 2:** Association between regional characteristics and age standardized incidence rates of thyroid cancer from 2009 to 2013 in Korea.

Prevalence (%)	Median	Range	Coefficient	Lower 95% CI	Upper 95% CI
Basic livelihood security recipient	3.7	0.3–12.8	− 1.59	− 2.51	− 0.67
High household income (≥ 500,000 won)	10.6	2.0–51.9	0.53	0.31	0.76
Heavy alcohol consumption	6.5	2.7–12.0	− 0.72	− 1.75	0.30
Heavy smoking	19.3	10.6–27.3	− 0.91	− 1.59	− 0.23
Moderate or vigorous PA (≥ 4 times per week)	5.5	0.6–18.8	0.23	− 0.27	0.72
Walking (≥ 4 times per week)	17.0	2.8–31.7	− 0.05	− 0.30	0.21
Obesity	22.1	14.6–30.7	− 0.10	− 0.72	0.51
Health checkup examinee within 2-year	58.8	49.3–70.7	− 0.05	− 0.41	0.31
Thyroid disorder diagnosis	1.8	0.1–4.4	3.24	1.47	5.00
Thyroid cancer screening examinee within 2-year	11.7	3.9–46.6	0.38	0.09	0.67

Heavy alcohol consumption indicates alcohol consumption ≥ 4 times a week. Heavy smoking indicates smoking ≥ 20 cigarettes per day. Obesity was defined as a body mass index of 25 kg/m^2^.

*CI* credible interval, *PA* Physical activity.

## Discussion

In this study, we found that the incidence of thyroid cancer was steadily higher in the southwest region compared with other regions. In addition, hot spots and cold spots for thyroid cancer incidence were confirmed in the northwest and northeast regions, respectively. These regional variations in thyroid cancer incidence can be partially explained by regional distribution of the prevalence of recipients of basic livelihood security, household income, smoking, thyroid dysfunction, and thyroid cancer screening.

The findings on the determinants of geographical variation in thyroid cancer incidence are quite plausible. We found that regions with a high prevalence of thyroid cancer screening were linked to high thyroid cancer incidence in this study. This finding is consistent with the findings of a previous study that reported a significant correlation between the regional prevalence of thyroid cancer screening and regional thyroid cancer incidence in Korea^14^. Province-level incidence of thyroid cancer was positively correlated with the regional prevalence of thyroid cancer screening.

Additionally, household income, a socioeconomic factor that affects the likelihood of undergoing thyroid cancer screening^16,17^, was associated with regional variations in thyroid cancer incidence in this study. Many studies have revealed that the epidemic of thyroid cancer is mainly impacted by overdiagnosis, that is, detection of small tumors through screening^3^. Although we could not directly evaluate the impact of overdiagnosis on regional variations in thyroid cancer incidence, our results suggest that some part of the spatial variation in thyroid cancer incidence was because of the regional difference in the prevalence of diagnosis.

Additionally, a 1% increase in heavy smoking prevalence was related to a decrease in approximately one thyroid cancer case per 100,000 in the present study. This finding is in line with those of previous studies that have reported a lower risk of thyroid cancer among smokers compared with non-smokers. A recent systematic review on the association between smoking and thyroid cancer incidence revealed that ever smoking was negatively associated with the risk of thyroid cancer (relative risk, 0.79, 95% confidence interval, 0.70 to 0.88)^18^. Although an inverse association between smoking and thyroid cancer risk has not been fully clarified, several mechanisms have been suggested as the link between smoking and lower risk of thyroid cancer. These mechanisms include reduction of the thyroid stimulating hormone (TSH) level^19,20^ and exertion of the anti-estrogenic effect^21,22^, conditions associated with the decrease in thyroid cancer risk^23,24^, because of smoking.

In addition, we found an evidence of positive association between the prevalence of thyroid dysfunction and thyroid cancer incidence. Although the role of thyroid dysfunction in thyroid cancer development is unclear, several mechanisms have been suggested as the link between functional thyroid disease and thyroid cancer. Thyroid dysfunction is a wide spectrum of diseases ranging from hypothyroidism to hyperthyroidism^25^; hence, previous studies have classified thyroid dysfunction into subtypes such as hypothyroidism and hyperthyroidism to evaluate the impact of each disease on thyroid cancer incidence^26–29^. In most previous studies, hyperthyroidism was significantly associated with an increased risk of subsequent thyroid cancer^27,28,30^. However, although a study reported hypothyroidism patients who are at a high risk for thyroid cancer^26^, hypothyroidism was not or less clearly associated with thyroid cancer incidence in these studies. One of the possible links between hyperthyroidism and increased thyroid cancer risk is the autoantibodies to the TSH receptor, which is the hallmark of Graves’ disease. Autoantibodies to TSH have been known to mimic the action of TSH^31^, which is linked to increased thyroid cancer risk^23^. In addition, hyperthyroidism has been reported to induce changes in sex hormones such as increase serum levels of estrogen and sex hormone-binding globulin and elevate the conversion ratio of testosterone to estradiol^32^, which are associated with the development of thyroid cancer^33^.

Although ionizing radiation from the environment has been suggested as a risk factor for thyroid cancer^34,35^, we found no significant association between the distance from nuclear power plants and thyroid cancer incidence rate in this study. This finding is contrary to the findings of a previous study that reported women residing within a < 5 km radius from the nuclear power plant as being at high risk of thyroid cancer in Korea^36^. This may be because of the loss of information; the distance from the radiation facility to the center of Sigungu district, rather than the actual distance from each residence to nuclear power plant was used for analyses. Therefore, it was difficult to evaluate the effect of external radiation from nuclear power plants on thyroid cancer incidence in this study clearly.

This study has several limitations. First, we performed an ecological study based on the Sigungu district-level information on disease outcomes and potential determinants. Inferences based on this study should be made carefully because this study is susceptible to the ecological inference fallacy that may arise when interpreting the results from aggregated data, unlike from individual data. Additionally, there would be a loss of useful information when using district-level aggregated data compared with individual-level data. Second, we could not consider the latency period of cancer development because the oldest information available on covariates preceding cancer incidence was the 2009 Korea Community Health Survey (KCHS) data. Third, other meaningful covariates on the spatial distribution of thyroid cancer incidence, such as exposure to medical radiation and iodine intake, were not considered. Fourth, detailed information on the classification of thyroid dysfunction and thyroid neoplasms was not obtained. In addition, previous studies have indicated that the effect of hyperthyroidism on thyroid cancer risk is quite different compared with that of hypothyroidism. Hence, if we could distinguish between hypothyroidism and hyperthyroidism cases, clearer results could have been obtained for the association between thyroid dysfunction and thyroid cancer. Although we could not consider the histological type of thyroid cancer, the most common histological type of thyroid cancer in Korea is papillary carcinoma (approximately 95% of all thyroid cancer cases)^37^; therefore, the results of this study can be said to be almost the same as those for the regional variation in papillary thyroid cancer and its determinants. If information on the histological type of thyroid carcinoma was available, we could have attempted to identify regional variations in each histological type of thyroid cancer and to evaluate the determinants of these variations. Fifth, information of thyroid cancer incidence after 2013 couldn’t be considered, thus we couldn’t reflect current situation to the analysis. However, it is unlikely that the results in this study will change significantly even if we considered recent information for the following reasons. Along with new guideline recommending not to use fine needle aspiration for thyroid cancer diagnosis in patient with thyroid nodule < 0.5 cm^38^ and evidence supporting the overdiagnosis of thyroid cancer in Korea^2,3^, the incidence of thyroid cancer between 2012 and 2016 showed a decreasing trend^39^. However, the recent thyroid cancer incidence in Korea is still higher than that of 10 years ago^1^. Furthermore, the facts that the evidences of overdiagnosis of thyroid cancer are steeply increased incidence restricted to papillary thyroid cancer and non-increasing mortality rate^14^, and the proportion of papillary thyroid cancer between 2012 and 2016 rather increased than that in the past indicate^39^ that overdiagnosis still exists widely in Korea. In addition, it is unlikely that there will be a sharp regional difference in the acceptance of thyroid cancer guidelines by physicians or the recognition of thyroid cancer overdiagnosis. Therefore, regional variation in thyroid cancer incidence would not change significantly after 2013. However, despite these limitations, this study has several strengths. One of the highlights is that this is the first national study to assess the spatial variations in thyroid cancer incidence and to identify determinants of variations based on spatial analysis using Bayesian inference in Korea. We considered spatial dependence between disease outcome and its determinants in the model to obtain less biased estimations through spatial analysis compared with those based on simple linear regression. Another strength of this study is that we used a nationwide representative database and uniform representative value of covariates. Information on all potential determinants of regional variation in thyroid cancer incidence was summarized as the regional proportion (%). Thus, it was quite intuitive and clear to interpret the association between regional variation in thyroid cancer incidence and its determinants by estimating the association between the increase in thyroid cancer incidence per 100,000 and 1% increase in each determinant.

In conclusion, this exploratory study is the starting point to better understand the regional variation in thyroid cancer incidence and its determinants based on spatial analysis in Korea. We identified the spatial clusters of thyroid cancer incidences in Korea and, thereafter, its determinants (such as basic livelihood security recipients, high household income, heavy smoking, thyroid dysfunction, and thyroid cancer screening) to explain the regional variation. We anticipate future studies to investigate the spatial epidemiology of thyroid cancer based on spatial analysis with individual point data of outcomes and potential determinants to overcome ecological bias. Furthermore, if subsequent studies undertake spatial analysis for other cancer types, as done in this study for thyroid cancer, their findings would lay the foundation for intervention and proper distribution of medical resources to target areas.

## Methods

We used the age-standardized incidence rate of thyroid cancer in the Sigungu district level reported by the National Cancer Center in 2016. The National Cancer Center provided district-level age-standardized incidence rates of 24 cancer types for three time periods (1999–2003, 2004–2008, and 2009–2013). Thyroid cancer was defined according to the International Classification of Diseases for Oncology third edition code of C73.

We used the administrative boundaries of 247 Sigungu districts in 2004 (one of the years in the second period of study) as the standard for regional boundaries. The administrative boundary shape file of the Sigungu districts was obtained from the Statistical Geographic Information Service (https://sgis.kostat.go.kr/). The district boundaries in 2004, the second study period (2004–2008), were used as the standard for regional boundaries; hence, separation and integration of Sigungu districts were performed in the first study period (1999–2003) and the third study period (2009–2013). In 2004, there were 247 Sigungu districts, and the cancer incidence rates in the first and third periods were modified to fit the format of 247 district-level cancer incidence rates in this study. In the case of integration of areas, we used the weighted average of the cancer incidence rates of the integrated areas. In the case of separation of an area, the cancer incidence rate of the area before separation was equally allocated to separate areas.

To account for regional variation in thyroid cancer incidence, we considered several covariates such as demographic factors, socioeconomic status factors, health behavior, and health care utilization, which are listed in Supplementary Table S2. We obtained covariate information from the KCHS to identify the determinants of regional variation in thyroid cancer incidence. Briefly, the KCHS is a nationwide community-based survey that was initiated with the purpose of providing well-grounded health services by producing representative health statistics in Korea^40^. The KCHS provides information on household surveys, health status and behavior, and diseases and injuries. We aggregated individual-level information of covariates to obtain the Sigungu district-level information by calculating the proportion of categorical variables (%). The KCSD was designed based on a multistage complex sampling method to achieve representative statistics of the entire Korean population. Therefore, we considered sampling components (strata and cluster) and appropriate weights in analyses to estimate the unbiased summary statistics of covariates in each Sigungu district. The PROC SURVEY procedure in the SAS program, which was developed for complex survey data analysis, was used in this study. Additionally, we considered the nearest distance from the nuclear power plant to the centroid of each Sigungu district as a covariate because radiation exposure is one of the major risk factors for thyroid cancer^41^. We converted the addresses of each nuclear power plant into geographic coordinates using geocoding. Thereafter, we calculated the nearest distance between each centroid point and nuclear power plant location based on the open source geographic information system software (QGIS™, version 3.10).

We identified spatial clusters with unusually high (hot spot) and low (cold spot) thyroid cancer incidence during three time periods (1999–2003, 2004–2008, and 2009–2013) based on the scan statistics of the normal model, available in SaTScan software™^42^. Spatial analysis based on scan statistics assesses the alternative hypothesis that the observations within one cluster come from a distribution different from the distribution on which the observations outside the cluster come from using the likelihood ratio test. The likelihood ratio test is repetitively performed by varying the location and size of the scanning window, which is the potential cluster across the study area, and the window with the maximum likelihood ratio is defined as the first cluster. In this study, spatial cluster analysis for the second cluster was performed for the region, except for the first cluster region. We set the maximum spatial scan window size to 50% of the population at risk. Age-standardized incidence rates and spatial clusters for thyroid cancer incidence were visualized on a map using QGIS™ (version 3.10).

A Bayesian hierarchical spatial model was employed to investigate the determinants of regional variation in thyroid cancer incidence in Korea. To identify determinants, data from 2009 to 2013 alone were analyzed because covariate information based on the KCSD was available from 2008. First, we evaluated the association between each covariate and thyroid cancer incidence using the linear regression model and selected covariates with a *P* value of < 0.2. Thereafter, we calculated variance inflation factors among selected covariates and accepted covariates with variance inflation factor values less than five to address multicollinearity. Finally, we included the proportion of following covariates in the model to identify the determinants of regional variation in thyroid cancer incidence: basic livelihood security recipients, high household income (≥ 5,000,000 won), heavy alcohol consumption (occasion ≥ 4-time per week), heavy smoking (≥ 20 cigarettes per day), moderate or vigorous physical activity (≥ 4-time per week), walking (≥ 4-time per week), obesity (body mass index ≥ 25 kg/m^2^), receiving health checkup within 2-year, thyroid dysfunction diagnosis, and receiving thyroid cancer screening within 2-year. We assessed the association between potential determinants and the ASR of thyroid cancer incidence by estimating the coefficient and two-tailed 95% credible intervals (95% CIs) with a significance level of 0.05. To consider spatial dependence in the neighborhood, we used the Besag–York–Mollie (BYM) model, which includes both an intrinsic conditional auto-regressive (ICAR) component for spatial auto-correlation and a random-effects component for non-spatial heterogeneity. The model is specified as in Eq. (1):

$${y}_{i}\sim Normal\left({\mu }_{i}, {\sigma }_{i}\right)$$

1 $${\mu }_{i}=\alpha +\sum_{k=1}^{K}{\beta }_{ik}{x}_{i}+{\nu }_{i}+{\upsilon }_{i}$$

$$\mu _{i} :linear\;prediction\;term;\;\alpha :intercept;\;\beta _{{ik}} :coefficient\;for\;each\;potential\;determinants;\;\nu _{i} :spatially\;unstructured\;random\;effects\;component;\;\upsilon _{i} :spatially\;structured\;component;\;i:each\;Sigungu\;district\;number;\;k:each\;covariate\;number\;included\;in\;the\;model$$

The spatial model was analyzed using the integrated nested Laplace approximation (ILNA), an alternative approach to the Monte Carlo Markov Chain, to reduce the computational cost of Bayesian inference. We assigned uninformative priors because of a lack of information on the distribution of each parameter. All data management and statistical analyses were performed using SAS software (version 9.4; SAS Institute Inc., Cary, NC, USA) and R software version 3.6.2, with the R-INLA package.

This study protocol was deemed exempt by the Institutional Review Board (IRB) of Korea University (IRB No. KUIRB-2021-0037-01).

## Supplementary Information

Supplementary Information.

## Data Availability

The datasets analyzed in the current study are publicly available from the Korean Statistical Information Service (https://kosis.kr) and the Korea Community Health Survey Data (https://chs.cdc.go.kr).
