# Supplementary material for: Spatial distribution and determinants of thyroid cancer incidence from 1999 to 2013 in Korea
Source: Sci Rep. 2021 Nov 18;11:22474. doi: 10.1038/s41598-021-00429-w (PMC8602462; doi:10.1038/s41598-021-00429-w)
Supplement: Supplementary file 1 — Supplementary Information. [file 41598_2021_429_MOESM1_ESM.docx]

Supplementary Table 1. Comparison of characteristics between regions inside and outside the thyroid cancer cluster during 2009–2013 in Korea

| Prevalence (%) | Inside cluster 1  (Hot spot) | Outside cluster 1 | *P* value | Inside cluster 2  (Cold spot) | Outside cluster 2 | *P* value |
| --- | --- | --- | --- | --- | --- | --- |
|  | Median (Range) | Median (Range) |  | Median (Range) | Median (Range) |  |
| Basic livelihood security recipient | 3.4 (0.3–12.8) | 3.4 (0.7–11.3) | 0.37 | 4.5 (1.6–9.5) | 3.4 (0.7–12.8) | <0.01 |
| High household income^1^ | 12.8 (2.0–51.9) | 9.9 (2.1–36.5) | <0.01 | 7.6 (2.7–14.5) | 11.6 (2.0–51.9) | <0.01 |
| Heavy alcohol drinking^2^ | 6.1 (2.7–10.5) | 6.8 (3.3–12.0) | <0.01 | 7.5 (4.4–12.0) | 6.3 (2.7–11.8) | <0.01 |
| Heavy smoking ^3^ | 18.1 (10.6–25.0) | 20.8 (14.7–27.3) | <0.01 | 21.6 (16.8–26.8) | 18.9 (10.6–27.3) | <0.01 |
| Moderate or vigorous physical activity^4^ | 6.1 (0.8–16.7) | 5.1 (0.6–18.8) | 0.01 | 4.3 (0.7–10.4) | 5.6 (0.6–18.8) | 0.01 |
| Walking^5^ | 16.2 (2.8–31.4) | 17.2 (4.6–31.7) | 0.38 | 16.4 (6.0–31.7) | 17.0 (2.8–31.4) | 0.61 |
| Obesity^6^ | 21.7 (14.6–29.1) | 22.7 (17.0–30.7) | <0.01 | 25.4 (19.2–30.7) | 21.8 (14.6–29.3) | <0.01 |
| Health checkup examinee | 58.6 (51.1–69.8) | 59.0 (49.3–70.7) | 0.42 | 62.1 (54.3–68.7) | 58.5 (49.3–70.7) | <0.01 |
| Thyroid dysfunction diagnosis | 1.8 (0.1–4.4) | 1.8 (0.6–4.4) | 0.85 | 1.7 (0.6–4.4) | 1.8 (0.1–4.4) | 0.56 |
| Thyroid cancer screening examinee | 13.6 (5.4–43.6) | 10.9 (3.9–46.6) | <0.01 | 10.6 (3.9–29.1) | 11.8 (4.6–46.6) | 0.37 |

1. Household income of ≥500,000 won
2. Alcohol drinking ≥4 times per week
3. Smoking ≥20 cigarettes per day
4. Performance of moderate and vigorous physical activity ≥4 times per week
5. Walking ≥4 times per week
6. Body mass index ≥25 kg/m^2^

Supplementary Table 2. List of covariates considered in analyses to identify determinants of spatial clusters of thyroid cancer

| Variables | Min^1^ | Median^1^ | Max^1^ | Year^2^ |
| --- | --- | --- | --- | --- |
| Male gender | 46.6 | 49.6 | 55.3 | 2009 |
| Married^3^ | 50.7 | 65.1 | 72.3 | 2009 |
| Education ≥12 years | 34.6 | 67.5 | 93.3 | 2009 |
| Basic livelihood security recipient | 0.3 | 3.7 | 12.8 | 2009 |
| High household income^4^ | 2.0 | 10.6 | 51.9 | 2009 |
| Cancer screening examinee | 24.9 | 45.9 | 65.1 | 2010 |
| Thyroid cancer screening examinee | 3.9 | 11.7 | 46.6 | 2010 |
| Diabetes mellitus diagnosis | 2.2 | 6.3 | 9.9 | 2009 |
| Thyroid dysfunction diagnosis | 0.1 | 1.8 | 4.4 | 2010 |
| Health checkup examinee | 49.3 | 58.8 | 70.7 | 2009 |
| Public health center usage^5^ | 7.7 | 27.2 | 82.4 | 2009 |
| Heavy alcohol drinking^6^ | 2.7 | 6.5 | 12.0 | 2009 |
| Heavy smoking^7^ | 10.6 | 19.3 | 27.3 | 2009 |
| Obesity^8^ | 14.6 | 22.1 | 30.7 | 2009 |
| Moderate or vigorous physical activity^9^ | 0.6 | 5.5 | 18.8 | 2009 |
| Walking ^10^ | 2.8 | 17.0 | 31.7 | 2009 |
| Distance from nuclear power plant to centroid of Sigungu district | 8.5 | 126 | 253 | 2009 |

Abbreviations: Min, Minimum value; Max, Maximum value

1. The unit of variables is percentage (%) except for the distance from nuclear power plant to centroid of Sigungu district (km)
2. Year of data collection
3. Living with a partner after marriage
4. Household income of ≥500,000 won
5. Visit to public health center at least once within the last year
6. Alcohol consumption ≥4 times a week
7. Smoking ≥20 cigarettes per day
8. Body mass index ≥25 kg/m^2^
9. Performance of moderate-intensity physical activity ≥4 times a week
10. Walking ≥4 times a week
